# BNPd single-atom catalysts for selective hydrogenation of acetylene to ethylene: a density functional theory study

**DOI:** 10.1098/rsos.171598

**Published:** 2018-07-25

**Authors:** Wanqi Gong, Lihua Kang

**Affiliations:** College of Chemistry and Chemical Engineering/ Key Laboratory for Green Processing of Chemical Engineering of Xinjiang Bingtuan, Shihezi University, Shihezi, Xinjiang 832000, People's Republic of China

**Keywords:** density functional theory, selective hydrogenation, B_11_N_12_Pd, single-atom catalyst, ethylene

## Abstract

The mechanisms of selective hydrogenation of acetylene to ethylene on B_11_N_12_Pd single-atom catalyst were investigated through the density functional theory by using the 6-31++G** basis set. We studied the adsorption characteristics of H_2_ and C_2_H_2_, and simulated the reaction mechanism. We discovered that H_2_ underwent absolute dissociative chemisorption on single-atom Pd, forming the B_11_N_12_Pd(2H) dihydride complex, and then the hydrogenation reaction with C_2_H_2_ proceeded. The hydrogenation reaction of acetylene on the B_11_N_12_Pd complex complies with the Horiuti–Polanyi mechanism, and the energy barrier was as low as 26.55 kcal mol^−1^. Meanwhile, it also has a higher selectivity than many bimetallic alloy single-atom catalysts.

## 1. Introduction

Ethylene is an important polymerization monomer and industrial reaction intermediate, and it is predominantly produced by the pyrolysis of hydrocarbons. The thermal cracking production process always contains on the order of 1% of acetylene; however, ethylene used in the production of polymers needs to contain less than a few parts per million of acetylene in order not to affect the polymerization process [1,2]. The most suitable way to remove a small amount of acetylene in ethylene is to catalyse the hydrogenation of acetylene to produce ethylene.

Pd catalysts have a good conversion rate for hydrogenation reactions and are used as a catalyst for the selective hydrogenation reaction [3–5]. However, the high price assumes far-reaching significance for the research and development of catalysts with high reactivity and selectivity [6,7]. The main reason for the poor selectivity is the comparable desorption and hydrogenation energy barrier of ethylene on the metal active sites [8]. The selectivity of the Pd catalysts can be improved by modifying with promoters, but this often adds some harmful metal or organic ligands [9–12]. Bimetallic alloys have been extensively explored as an alternative to Pd catalysts [13–17]. But, this technique is limited by the electronic properties of the two metals that are easily separated and inactivated at high temperatures. In order to improve the stability of bimetallic alloys, it is a useful measure to alloy the metal with another metal to form an alloyed single-atom catalyst (SAC) [18,19]. A growing number of reports suggest that Pd atom isolation is beneficial to the selectivity [20–22]. Meanwhile, Zhang and colleagues demonstrated that maximum reduction of the metal particles can not only improve the utilization of metal atoms, but also enhance the catalytic efficiency and selectivity [23,24]. Some of the literature suggests that the selectivity of IB metal alloyed Pd SACs to ethylene might be affected by electron transfer between the IB metal and Pd atom, through DFT calculation [5,25,26]. Lu and colleagues proved that Pd_1_/C_3_N_4_ SAC can enhance both selectivity and coking-resistance for acetylene hydrogenation [27]. This encouraged us to explore single-atom catalysts for hydrogenation of acetylene. In order to obtain a stable single-atom catalyst, we chose a B_12_N_12_ nanocage as the support and inserted Pd into the B_12_N_12_ framework.

Since Kroto and colleagues discovered C_60_ in 1985, many researchers have further explored fullerene and related materials [28]. As the analogues of carbon fullerenes, boron nitride (BN) nanocage clusters have been successfully synthesized [29–31], Oku and colleagues successfully synthesized the B_12_N_12_ cage and revealed that its four- and six-membered BN rings satisfy the isolated tetragonal rule [30,32]. Among these doped fullerenes, the B_12_N_12_ cage is considered to be the smallest stable cage [30,31,33]. Researchers mainly explored these clusters as catalysts on their application as hydrogen-storage materials [34–37]. In this work, a doped B_12_N_12_ cage as a B_12_N_11_Pd SAC was used as a catalyst for selective hydrogenation of acetylene to ethylene for the first time.

We use density functional theory (DFT) to study the adsorption of H_2_ and C_2_H_2_ on B_12_N_11_Pd SAC. The results show that a Pd single atom can effectively dissociate an H_2_ molecule, which is consistent with the demonstration of Sykes and co-workers [19,38,39]. From the selectivity formula, we conclude that the selectivity of the B_11_N_12_Pd SAC is higher than that of the majority of the bimetallic alloy single-atom catalysts [16,17,26]. Our work indicated that the B_11_N_12_Pd SAC might be a promising candidate for selective hydrogenation reactions.

## 2. Calculation method and models

All density functional calculations were executed using the Gaussian09 program package [40]. The hybrid density functionals of Lee, Yang and Parr (B3LYP) with the 6-31++G** basis set were applied for all structures. The nonlocal correlation functional of B3LYP [41] with the 6-31++G** basis set was used for H, C, B and N atoms, and the B3LYP functional was combined with the LANL2DZ basis set for the Pd atoms. No symmetry constraints were imposed on the geometry optimization. All relative energies in this study were zero-point-energy. All stationary points were characterized as the minima (no imaginary frequency) or transition state (TS; one imaginary frequency) by Hessian calculation. Intrinsic reaction coordinate (IRC) calculations [42,43] were performed to determine if each TS links the correct product with the reactant. Transition-state structures were characterized using frequency calculations and by analysing the vibrational modes. In all instances, only one imaginary frequency corresponding with the reaction coordinate was obtained.

An essential reference point for this calculation is the adsorption energy for C_2_H_2_ and H_2_ absorbed on the B_11_N_12_Pd nanocage. In this paper, we used the following definitions for adsorption energy.

When H_2_ or C_2_H_2_ is absorbed on B_11_N_12_Pd-SAC, the adsorption energy calculated was defined by the following equation.

2.1 $$E=E_{\mathrm{total}}-E_{B_{11}N_{12}\mathrm{Pd}}-E_{H_{2}/C_{2}H_{2}}.$$

When H_2_ is absorbed on BNPd-C_2_H_2_, the adsorption energy is calculated as the following equation:

2.2 $$E=E_{\mathrm{total}}-E_{B_{11}N_{12}\mathrm{Pd}-C_{2}H_{2}}-E_{H_{2}}.$$

When C_2_H_2_ is absorbed on HCl, the adsorption energy is calculated as the following equation:

2.3 $$E=E_{\mathrm{total}}-E_{B_{11}N_{12}\mathrm{Pd}-H_{2}}-E_{C_{2}H_{2}}.$$

When H_2_ and C_2_H_2_ are co-absorbed on B_11_N_12_Pd SAC, the co-adsorption energy is calculated as the following equation.

2.4 $$E=E_{\mathrm{total}}-E_{B_{11}N_{12}\mathrm{Pd}}-E_{C_{2}H_{2}}-E_{H_{2}},$$

*E*_total_ is the total energy of the absorption system; $E_{B_{11}N_{12}\mathrm{Pd}}$ is the energy of the B_11_N_12_Pd SAC. $E_{H_{2}/C_{2}H_{2}}$ is the energy of H_2_ or C_2_H_2_. $E_{B_{11}N_{12}\mathrm{Pd}-H_{2}}$ is the energy of the C_2_H_2_ adsorbed on the BNPd nanocage single-atom catalyst.

## 3. Results and discussion

### 3.1. Optimized B_11_N_12_Pd

**Figure 1.**
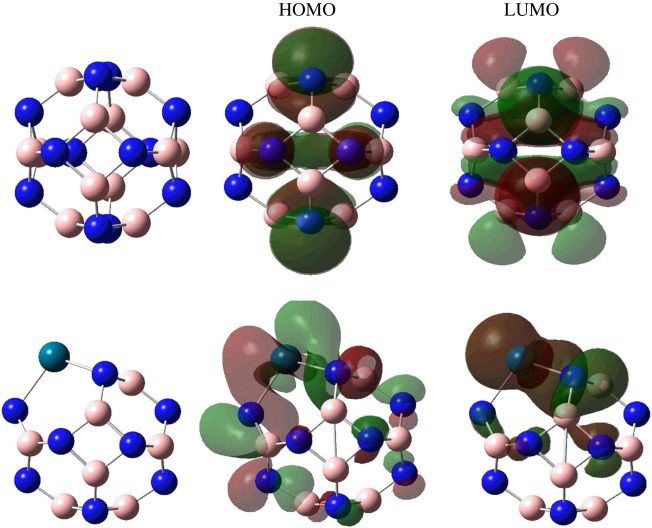
The optimized structures of undoped B_12_N_12_ cluster and Pd-doped B_11_N_12_Pd SAC and the corresponding calculated HOMO and LUMO at B3LYP/6-31++G** level. H, white; B, pink; N, blue; Pd, blue-green.

The optimized structures of the pristine B_12_N_12_ and B_11_N_12_Pd are depicted in figure 1. As expected, the calculated result shows that the charges uniformly distribute among all B(N) atoms in B_12_N_12_, with a Mulliken value of 0.57 e for B, and −0.57 e for N, which is consistent with previous calculations [36]. While one of the B-N bonds was shared between two six-membered rings with a length of about 1.44 Å, the other was shared between a four- and a six-membered ring with length of 1.48 Å. By substituting one Pd atom for a B site in the B_12_N_12_ nanocage, the charges redistribute on the B_12_N_12_ cage, and the electrons are found to accumulate around the Pd atom in B_11_N_12_Pd. The Mulliken charge for Pd is about 2.22 e, while the charge of the replaced B was 0.57 e, which means that the electron transfer is 1.65 e from the Pd atom to the B_12_N_12_ nanocage. The Pd-N bond which was shared between the two six-membered rings had a length of about 2.10 Å, while the other was shared between a four- and a six-membered ring with a length of 2.04 Å. The adjacent three N atoms have a Mulliken charge of −0.37, −0.37 and −0.38 e. This means that the Pd atom and BN nanocage had a strong interaction.

In table 1, we list the HOMO and LUMO distributions and frontier molecular orbital (FMO) energies of the B_12_N_12_ and B_11_N_12_Pd nanocages, respectively. One can see that after Pd doping, the orbital energy of HOMO was reduced, which means the stability of the B_11_N_12_Pd SAC is enhanced; the band gap (Δ*E*_g_) between HOMO and LUMO also decreases, which facilitates the interaction between adsorbates and the surfaces.

**Table 1.** The orbital energies for the HOMO and LUMO of H_2_, C_2_H_2_, B_12_N_12_ and B_11_N_12_Pd SAC, and their energy gaps (△*E*_g_) between H_2_, C_2_H_2_ and B_12_N_12_/B_11_N_12_Pd. Energies are in eV.

				HOMO–LUMO			
	HOMO	LUMO	△*E*_g_	(H_2_ → BN)	(BN → H_2_)	(C_2_H_2_ → BN)	(BN → C_2_H_2_)
H_2_	11.83	0.53					
C_2_H_2_	−8.08	0.11					
B_12_N_12_	7.95	−1.24	6.72	−10.59	−8.49	−6.85	−8.06
B_11_N_12_Pd	−6.64	−4.33	2.31	−7.50	−7.17	−3.75	−6.75

### 3.2. Adsorption of reactants

**Figure 2.**
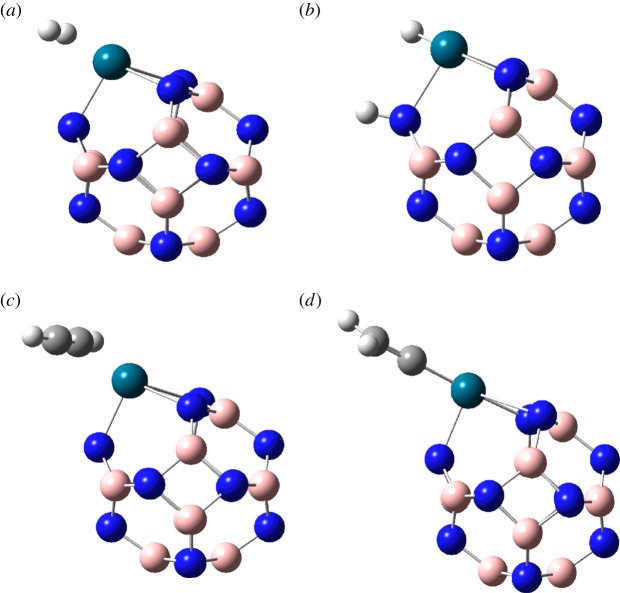
The stable structures of H_2_ and C_2_H_2_ absorbed on B_11_N_12_Pd SAC. H, white; B, pink; N, blue; Pd, blue-green.

The molecular orbital calculations and electron density analysis can be used to determine the adsorption position of C_2_H_2_ and H_2_ molecules on B_11_N_12_Pd SAC. According to the FMO analysis of the B_11_N_12_Pd SAC, the Pd atom may be the only active site (figure 1). In order to fully consider the possible adsorption sites, we put C_2_H_2_ and H_2_ on different sites both around the Pd atom and the BN nanocage structure, and obtained the most stable adsorption structures, which are shown in figure 2.

**Figure 3.**
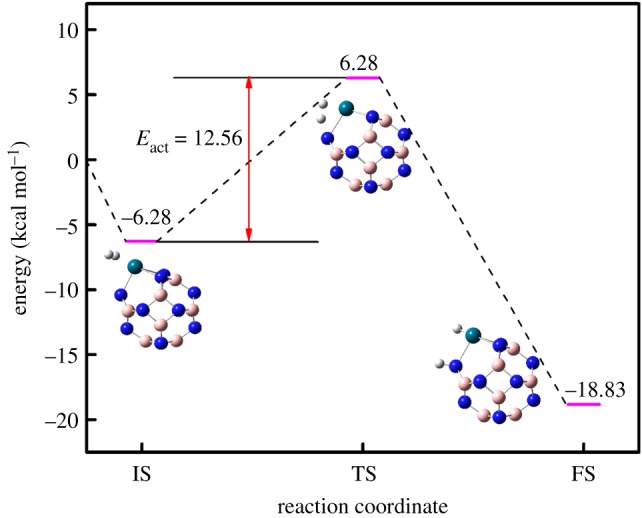
Calculated hydrogenation reaction diagram for the B_11_N_12_Pd SAC. The schematic diagrams of initial (IS), transition (TS) and final (FS) states are illustrated by side views. H, white; B, pink; N, blue; Pd, blue-green.

Figure 3 shows that an H_2_ molecule was initially absorbed on the Pd atom of the B_11_N_12_Pd SAC with an adsorption of about 6.28 kcal mol^−1^; the length between Pd and H_2_ was about 1.94 Å, the bond length of H_2_ was 0.78 Å, and it increased by 0.04 Å compared with the free H_2_ molecule, indicating that there was interaction between H_2_ and B_11_N_12_Pd SAC. Subsequently, a TS was formed when the H–H bond length was about 1.01 Å, which increased by 0.27 Å compared with the free H_2_ molecule. From the vibrational analysis, we obtained only one imaginary frequency (−1426.63 cm^−1^), as the two H atoms of H_2_ gradually separate approaching the B and Pd atoms. Then the two H atoms finally settled and formed the B_11_N_12_Pd(2H) dihydride complex, with a 2.72 Å distance for H–H, and the system gains a stabilization energy of −18.83 kcal mol^−1^, indicating H_2_ absolutely dissociative chemisorption.

To investigate the kinetic issue in the process of the hydrogenation on the B_11_N_12_Pd SAC, we calculated the activation barrier for one H_2_ fully chemisorbed on the B_11_N_12_Pd SAC. Figure 3 shows the calculated reaction diagram for the H_2_ adsorption on B_11_N_12_Pd SAC. The corresponding activation barrier (energy difference between TS and initial state (IS)) for the hydrogenation reaction is found to be 12.56 kcal mol^−1^, indicating that H_2_ dissociative chemisorption on the B_11_N_12_Pd SAC and the diffusion process of H atoms are facile.

In table 2, the Mulliken charge of absorbed C_2_H_2_ is 0.47 e, indicating that an electron transfer of 0.47 e occurred from C_2_H_2_ to B_11_N_12_Pd SAC; the length between Pd and C_2_H_2_ is 2.30 Å, meaning that the C_2_H_2_ molecules were absorbed only on the single Pd atom. The optimal adsorption energy of C_2_H_2_ is −14.95 kcal mol^−1^, which can be seen in table 2.

**Table 2.** The optimal adsorption energies and Mulliken charge of H_2_, C_2_H_2_, C_2_H_4_, C_2_H_6_ separately absorbed on B_11_N_12_Pd SAC. H + H represents the H_2_ dissociative adsorption on B_11_N_12_Pd SAC. Energies are in kcal mol^−1^; Mulliken charges are in *e.*

	energies	Mulliken charge
H_2_	−3.32	0.24
H + H	−18.83	0.45
C_2_H_2_	−14.95	0.47
C_2_H_4_	−16.55	0.26
C_2_H_6_	−2.41	−0.02

**Table 3.** Comparison of the energy barriers for the desorption energy of ethylene (*E*_a1_), desorption barriers of ethylene (*E*_ad)_ and the difference between desorption and hydrogenation barrier of ethylene (*ΔE*_a_) with different catalysts. Energies are in kcal mol^−1^.

	*E*_a1_	*E*_ad_	*ΔE*_a_	reference
B_11_N_12_Pd	29.35	−16.55	12.8	this work
Au-Ni(111)	13.84	−6.23	7.61	16
Cu-Ni(111)	16.6	−14.07	2.54	16
Ag-Cu(111)	15.91	−6.46	9.45	17
Pd-Cu(000)	17.29	−11.07	6.22	17
PdGa	21.22	−11.99	9.23	23

### 3.3. Mechanisms of selective hydrogenation of acetylene to ethylene by B_11_N_12_Pd single-atom catalyst

#### 3.3.1. C_2_H_2_ adsorbed onto the B_11_N_12_Pd(2H) dihydride complex

**Figure 4.**
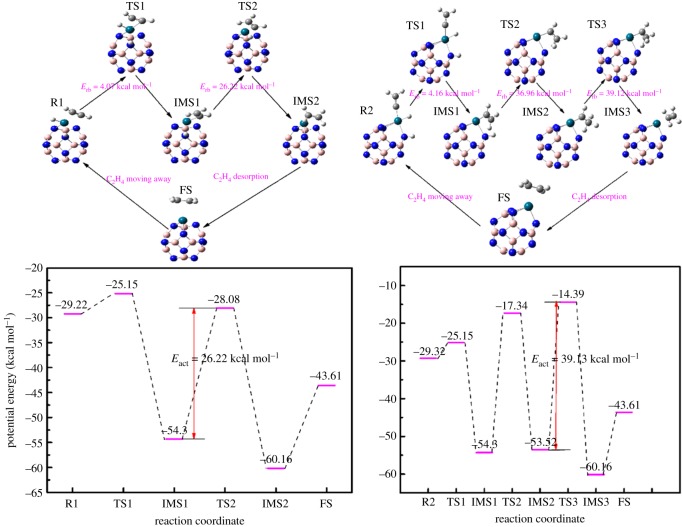
The reaction process and potential energy change for the different reaction pathways R1 (left) and R2 (right) of catalytic hydrogenation of acetylene to ethylene on the B_11_N_12_Pd SAC. The schematic diagrams of co-adsorption (R), transition (TS), intermediate (IMS) and final (FS) states are illustrated by side views. H, white; B, pink; N, blue; Pd, blue-green; C, grey.

As shown in figure 4 (R1), the B_11_N_12_Pd(2H) dihydride complex adsorbed by the C_2_H_2_ is a little different from that on the bare B_11_N_12_Pd SAC. Simultaneously, the binding sites of H atoms on B_11_N_12_Pd(2H) dihydride complex are practically unchanged. When C_2_H_2_ is absorbed on B_11_N_12_Pd(2H) dihydride complex as equation (2.3), the adsorption energy is calculated to be −11.98 kcal mol^−1^, indicating that the complex has a high adsorption to C_2_H_2_. The most stable adsorption energies of H_2_ and C_2_H_2_ on the B_11_N_12_Pd SAC are −17.23 and −14.95 kcal mol^−1^, respectively, as shown in equation (2.1). As in equation (2.4), the co-adsorption of H_2_ and C_2_H_2_ on the B_11_N_12_Pd SAC is −29.22 kcal mol^−1^, which is less than the sum of the individual C_2_H_2_ and H_2_ adsorption energies (−32.18 kcal mol^−1^). This indicates that there is an interaction between H_2_ and C_2_H_2_, and the adsorption of the two H atoms does not change obviously when the two adsorbates occur simultaneously on the B_11_N_12_Pd SAC. The results show that this interaction does not affect the mechanism of the reaction.

As figure 4 shows, the complex of acetylene (R1) is formed by the adsorption of the acetylene molecule on B_11_N_12_Pd(2H). Both of the reactants were absorbed on the single Pd atom, which can provide an excellent condition for heterogeneous catalytic reactions. R1 passes through a small energy barrier to obtain TS1. From the vibrational analysis of TS1, we obtained only one imaginary frequency (−645.43 cm^−1^), which was associated with the stretching motion of the H atom adjacent to the Pd atom. We can deduce that the H atom adjacent to Pd is active in TS1. IRC calculation testified that TS1 connected the co-adsorption and IMS1 and showed that no further intermediates are involved in the reaction. Then the H atom that is linked to the Pd atom approaches to form a vinyl-B_11_N_12_Pd(H) intermediate (IMS1); the length of C=C double bonds is 1.34 Å, which is close to the C=C bond length of free C_2_H_4_. The H atom which is linked with the N atom has a nearly neutral charge, which denotes the weak binding of intermediates. The IMS1 through a 26.22 kcal mol^−1^ energy can proceed to the TS2; from the vibrational analysis of TS2, we obtained only one imaginary frequency (−1041.20 cm^−1^), which was associated with the stretching motion of the H atom linked to the B atom. IMS2 approaches the vinyl-B_11_N_12_Pd and forms an ethane molecule on the B_11_N_12_Pd SAC. Then C_2_H_4_ is desorbed and achieves the final state (FS); the C_2_H_4_ only needs 16.55 kcal mol^−1^ energy to move away, and the low desorption energy can ensure the selectivity of ethylene.

From the energy diagrams of the R1, the step from IMS1 to TS2 is the rate-limiting step. We calculated the activation energy of hydrogenation of acetylene to ethylene action as 26.22 kcal mol^−1^ (the activation energy is the biggest energy difference in the energy diagram, as shown in figure 4), which is similar to the Pd_5_ cluster (the lowest activation energy is 25.72 kcal mol^−1^) [44]. This indicates that the B_11_N_12_Pd SAC has a similar activation energy for hydrogenation of acetylene to ethylene; moreover, it can make full use of the noble metal and decrease the price of the catalyst.

#### 3.3.2. Vinylidene adsorbed onto the B_11_N_12_Pd(2H) dihydride complex

As figure 4 (R2) shows, there is another pathway from vinylidene (C=CH_2_) to C_2_H_4_, in which the H atom adsorbed on the Pd atom of R2 can move to the vinylidene to form ethenyl-B_11_N_12_Pd(H), with only 4.16 kcal mol^−1^ difference of free energies. Then the remaining H atom which linked to the B atom of IMS1 is transferred to the ethenyl group to obtain ethylidene via TS2, whose free energy is −36.96 kcal/mol. In the complicated pathways of acetylene hydrogenation, ethylidene can obtain the C_2_H_4_ by proton translocation (TS3). The free energies of the TS for the IMS2 and IMS3 are −39.12 kcal mol^−1^. The final step was the C_2_H_4_ molecule desorption from the B_11_N_12_Pd, and the desorption energy was 16.55 kcal mol^−1^. From the vibrational analysis of the TS, we obtained only one imaginary frequency for each TS (−1099.77, −1167.07, −923.56 cm^−1^). To gain a better understanding of the reaction, series IRC calculation testified that the TS connected the co-adsorption and IMS, and showed that no further intermediates are involved in the reaction.

From the potential energy change of catalytic hydrogenation of acetylene to ethylene in figure 4 (R2), the activation energy is 39.12 kcal mol^−1^, and the rate-controlling step is also from IMS2 to TS3. Considering the complex hydrogenation pathways, it is found that the semi-hydrogenation of acetylene on the B_11_N_12_Pd cluster is easy to achieve.

#### 3.3.3. H_2_ adsorbed onto the B_11_N_12_Pd–C_2_H_2_ complex

We also investigated the non-Horiuti–Polanyi mechanism of selective hydrogenation of acetylene on the B_11_N_12_Pd, as shown in the electronic supplementary material, figure S1–S3; the activation energy of hydrogenation of acetylene to ethylene action is 57.79, 53.26 and 55.84 kcal mol^−1^. This means that selective hydrogenation of acetylene on the B_11_N_12_Pd complies with the Horiuti–Polanyi mechanism. Therefore, the lowest activation energy of hydrogenation of acetylene to ethylene is 26.22 kcal mol^−1^.

#### 3.3.4. C_2_H_4_ adsorbed onto the B_11_N_12_Pd(2H) dihydride complex

**Figure 5.**
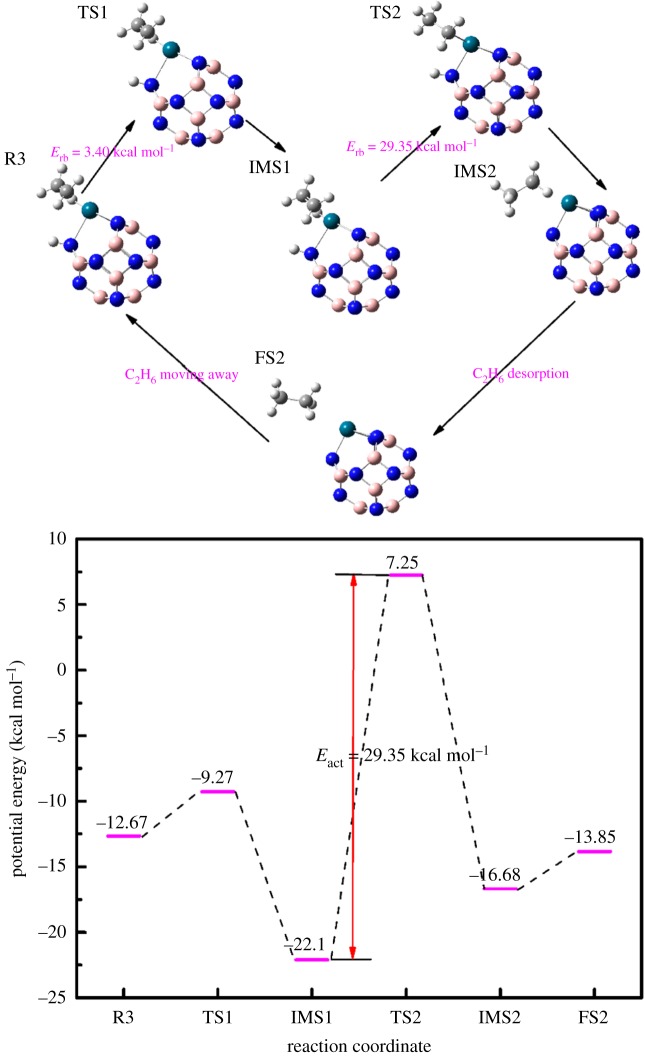
The reaction process and potential energy change of catalytic hydrogenation of ethylene to ethane on the B_11_N_12_Pd SAC. The schematic diagrams of co-adsorption (R), transition (TS), intermediate (IMS) and final (FS) states are illustrated by side views. H, white; C, grey; B, pink; N, blue; Pd, blue-green.

In order to clearly research the selectivity of the catalyst, we studied the reaction of ethylene to produce ethane. As figure 5 shows, C_2_H_4_ was first adsorbed onto the B_11_N_12_Pd(2H) dihydride complex to form the co-adsorption configuration (R3); according to equation (2.3) the adsorption energy is −12.67 kcal mol^−1^, indicating that the complex also has a high adsorption to C_2_H_4_ compared with C_2_H_2_. R3 passes through a small energy barrier to obtain TS1. From the vibrational analysis of TS1, we obtained only one imaginary frequency (−639.02 cm^−1^), which was associated with the stretching motion of the H atom that is linked to the Pd atom. We can observe that the H atom adjacent to Pd is active in TS1. Then the H atom approaches to form an ethyl-B_11_N_12_Pd(H) intermediate (IMS1); the length of C=C double bonds of C_2_H_4_ is 1.37 Å, which is raised 0.03 Å compared with the TS1. The IMS1 through a 29.35 kcal mol^−1^ energy can proceed to the TS2.

IRC calculation testified that TS1 connected the co-adsorption and IMS1 and showed that no further intermediates are involved in the reaction. The length of C=C double bonds is 1.34 Å, which is close to the C=C bond length of free C_2_H_4_. The H atom which is linked with the N atom has a nearly neutral charge, which denotes the weak binding of intermediates. The IMS1 through a 26.22 kcal mol^−1^ energy can proceed to the TS2; from the vibrational analysis of TS2, we obtained only one imaginary frequency (−1597.33 cm^−1^), which was associated with the stretching motion of the H atom linked to the B atom. IMS2 approaches the vinyl-B_11_N_12_Pd and forms an ethane molecule on the B_11_N_12_Pd SAC. Then C_2_H_6_ is desorbed and the FS is obtained; the dissociation energy of C_2_H_6_ is about 2.83 kcal mol^−1^.

From the energy diagrams of R3, the step from IMS1 to TS2 is the rate-limiting step. We calculated the activation energy of hydrogenation of ethylene to ethane action as 29.35 kcal mol^−1^, which is higher than that of the acetylene to ethylene action. This indicates that the B_11_N_12_Pd SAC has a high selectivity of acetylene hydrogenation to ethylene.

#### 3.3.5. H_2_ adsorbed onto the B_11_N_12_Pd–C_2_H_4_ complex

We also investigated the hydrogenation of ethylene to ethane action onto the B_11_N_12_Pd in non-Horiuti–Polanyi mechanism (electronic supplementary material, figure S4); the activation energy is 53.11 kcal mol^−1^. This means that hydrogenation of ethylene onto the B_11_N_12_Pd complies with the Horiuti–Polanyi mechanism. Therefore, the lowest activation energy of hydrogenation of acetylene to ethylene is 29.35 kcal mol^−1^.

### 3.4. Selectivity of the acetylene hydrogenation to ethylene on B_11_N_12_Pd SAC

The factor influencing the selectivity of acetylene hydrogenation to ethylene is considered to be the difference between the desorption barriers and the hydrogenation barrier of ethylene [8,45]; we defined the difference as $\Delta E_{a}$. The desorption barriers are estimated with the absolute value of the adsorption energies according to the approximation made in previous studies [8,16,46–50]. Namely, we define

3.1 $$\Delta E_{a}=E_{a}-|E_{ad}|,$$

where *E_a_* and $E_{ad}$ are the hydrogenation and desorption barriers of ethylene, respectively. This equation indicates that the more positive the Δ*E*_a_, the more selective the catalyst will be for the production of ethylene compared with ethane formation. As table 1 shows, B_11_N_12_Pd has a better selectivity than most bimetallic alloys [13,16,17,26].

A high selectivity also can be judged from the following two aspects. One is a low desorption energy of ethylene, which can effectively inhibit the hydrogenation of ethylene to produce ethane. In this work, the desorption energy of ethylene (16.55 kcal mol^−1^) is less than the activation energy of ethylene (29.35 kcal mol^−1^), which can ensure a high selectivity. The other is a high ethylene hydrogenation activation energy. In this work, the acetylene hydrogenation activation energy is 26.55 kcal mol^−1^, which is less than the ethylene hydrogenation activation energy. Therefore, we can draw the conclusion that the B_11_N_12_Pd SAC has a high selectivity for the acetylene hydrogenation to ethylene.

## 4. Conclusion

This work uses DFT to study the catalytic process of selective hydrogenation on B_11_N_12_Pd SAC. The results show that a Pd single atom can effectively dissociate an H_2_ molecule and form the B_11_N_12_Pd(2H) dihydride complex, with the hydrogenation of acetylene to ethylene following the Horiuti–Polanyi mechanism. The activation energy for hydrogenation of acetylene is similar to that with Pd clusters, which is beneficial to reduce the cost of the catalyst. Besides, the low desorption energy of ethylene and high ethylene hydrogenation activation energy can ensure that the B_11_N_12_Pd SAC has a high selectivity. From the selectivity formula, we conclude that the selectivity of the B_11_N_12_Pd SAC is higher than that of the majority of the binary metal monatomic catalysts. This work provides a theoretical basis for the development of catalysts with novel high catalytic performance and selectivity for hydrogenation.

## Supplementary Material

Cartesian coordinates

## Data accessibility

All original data can be accessed at the Dryad Digital Repository [51].
