# Supplementary material for: BNPd single-atom catalysts for selective hydrogenation of acetylene to ethylene: a density functional theory study
Source: R Soc Open Sci. 2018 Jul 25;5(7):171598. doi: 10.1098/rsos.171598 (PMC6083668; doi:10.1098/rsos.171598)
Supplement: Cartesian coordinates [file rsos171598supp1.docx]

**BNPd Single-atom Catalysts for Selective Hydrogenation of Acetylene to Ethylene: A DFT Study**

Wanqi Gong, Lihua Kang*

College of Chemistry and Chemical Engineering/ Key Laboratory for Green Processing of Chemical Engineering of Xinjiang Bingtuan. Shihezi University, Shihezi, Xinjiang, 832000, PR China

Fig. S1 The reaction process and potential energy change of catalytic hydrogenation of acetylene to ethylene on the B_11_N_12_Pd SAC. The schematic diagrams of co-adsorption (R), transition (TS), intermediate (IMS) and final (FS) states are illustrated by side views. H, white; C,gray; B, pink; N, blue; Pd, blue-green.


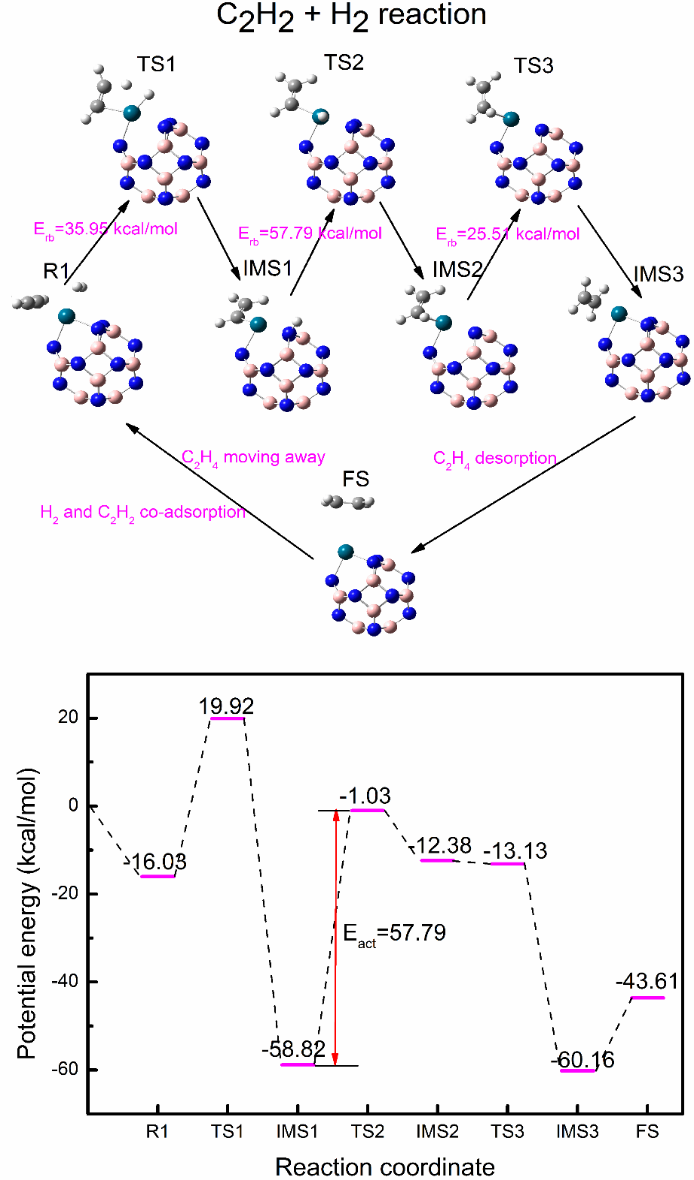


Fig. S2 The reaction process and potential energy change of catalytic hydrogenation of vinylidene to ethylene on the B_11_N_12_Pd SAC. The schematic diagrams of co-adsorption (R), transition (TS), intermediate (IMS) and final (FS) states are illustrated by side views. H, white; C,gray; B, pink; N, blue; Pd, blue-green.


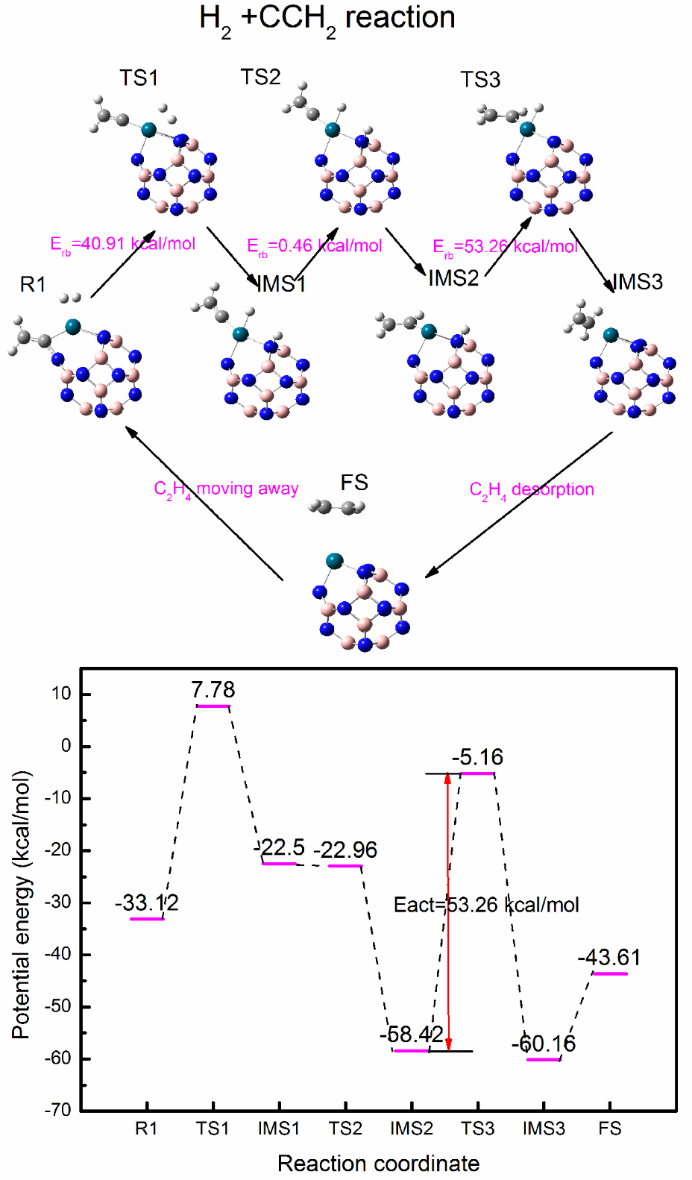


Fig. S3 The reaction process and potential energy change of catalytic hydrogenation of acetylene to ethylene on the B_11_N_12_Pd SAC. The schematic diagrams of co-adsorption (R), transition (TS), intermediate (IMS) and final (FS) states are illustrated by side views. H, white; C,gray; B, pink; N, blue; Pd, blue-green.


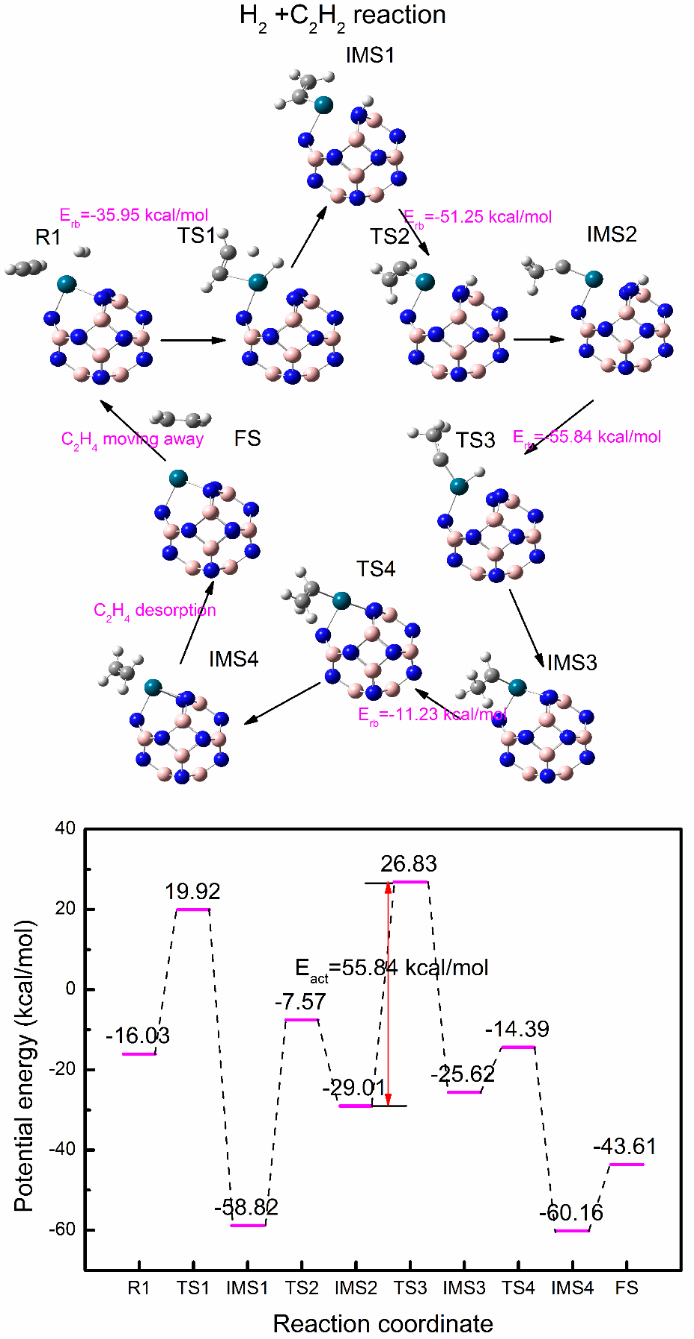


Fig. S4 The reaction process and potential energy change of catalytic hydrogenation of ethylene to ethane on the B_11_N_12_Pd SAC. The schematic diagrams of co-adsorption (R), transition (TS), intermediate (IMS) and final (FS) states are illustrated by side views. H, white; C,gray; B, pink; N, blue; Pd, blue-green.


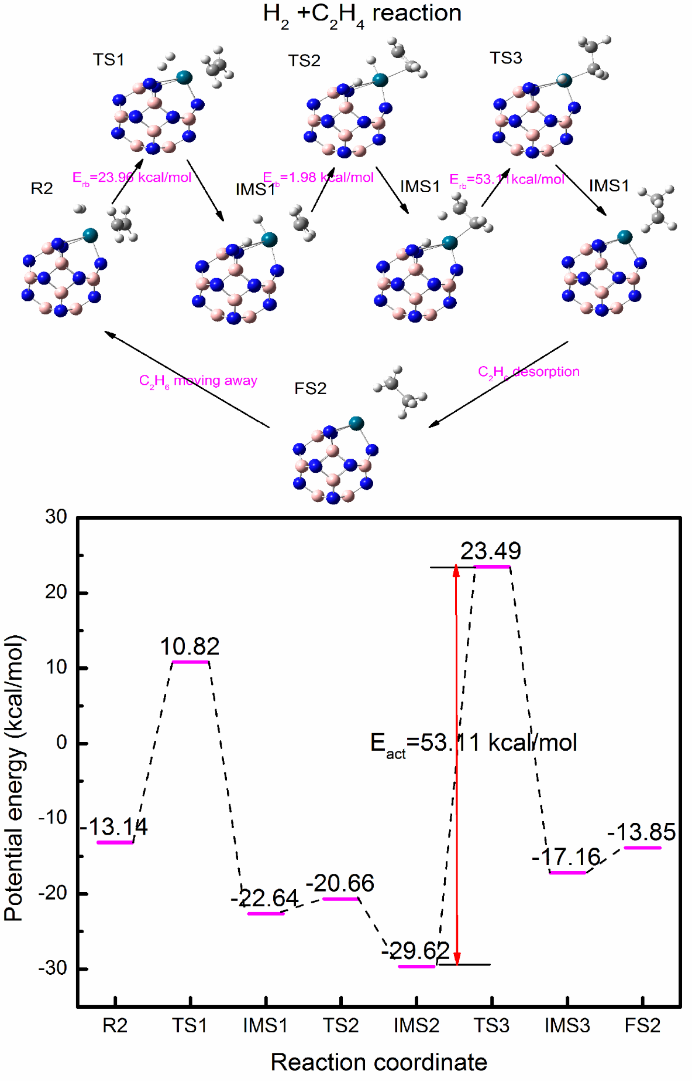


**Cartesian coordinates [in B3LYP/6-31++G(d, p) level of theory] for the key structures involved in this study.**

Figure 1

B_12_N_12_

B -0.00000000 0.96135307 1.97139615

B -0.00000000 -0.96135307 1.97139615

B -1.97139615 0.00000000 0.96135307

B -0.96135307 1.97139615 0.00000000

B 1.97139615 0.00000000 0.96135307

B 0.96135307 1.97139615 -0.00000000

B 0.00000000 0.96135307 -1.97139615

B 1.97139615 -0.00000000 -0.96135307

B -0.00000000 -0.96135307 -1.97139615

B -0.96135307 -1.97139615 0.00000000

B 0.96135307 -1.97139615 -0.00000000

N -1.12361309 0.00000000 2.12435116

N -0.00000000 2.12435116 1.12361309

N -2.12435116 1.12361309 -0.00000000

N -2.12435116 -1.12361309 0.00000000

N -0.00000000 -2.12435116 1.12361309

N 2.12435116 1.12361309 -0.00000000

N 0.00000000 2.12435116 -1.12361309

N 1.12361309 0.00000000 -2.12435116

N -1.12361309 -0.00000000 -2.12435116

N -0.00000000 -2.12435116 -1.12361309

N 2.12435116 -1.12361309 0.00000000

B -1.97139615 0.00000000 -0.96135307

N 1.12361309 -0.00000000 2.12435116

B_11_N_12_Pd

Energy=-1057.783864 (a.u.)

B -0.23546400 1.86761100 -0.92299100

B -0.23573400 1.86642100 0.92477700

B -0.15798500 -0.46726600 -1.88653600

B 2.03225700 1.78937100 0.00111300

B 1.52741500 0.45361800 -1.92337800

B 1.66042400 -1.75024300 -0.95635600

B 2.91299000 0.07860200 0.00025300

B 1.66030300 -1.75136600 0.95459300

B -0.15833500 -0.46971700 1.88592200

B 1.52702400 0.45139500 1.92396100

N 0.13442000 0.97186300 -2.00694800

N -1.17563200 -1.20594200 -1.17300500

N -1.17592000 -1.20768600 1.17165700

N 0.13395700 0.96939400 2.00772600

N 2.59180100 0.99887200 -1.12350800

N 1.22682900 -0.98760400 -2.09820000

N 2.72576300 -1.34743800 -0.00058300

N 0.76170400 -2.44052900 -0.00135700

N 1.22654100 -0.99003300 2.09724700

N 2.59159000 0.99761200 1.12497300

B -0.55269800 -1.83318600 -0.00106100

N 0.77195100 2.45957900 0.00137200

N -1.37590800 1.82568200 0.00073700

Pd -2.36871000 -0.03223000 -0.00004900

Figure 2

a (B_11_N_12_Pd-H_2_)

B -0.22220400 2.02955900 -0.19150900

B -0.23597300 1.23743300 1.51710800

B -0.06575800 0.31693300 -1.91112300

B 2.01697300 1.69474100 0.70436500

B 1.59047900 1.22655400 -1.61413400

B 1.77944300 -1.18140800 -1.53567700

B 2.98283400 0.16985400 0.05029900

B 1.82017800 -1.93822200 0.22282300

B -0.02915000 -1.28103900 1.59893500

B 1.60964900 -0.31099400 1.96123000

N 0.19052200 1.72313700 -1.53870900

N -1.04055700 -0.60725100 -1.40746100

N -1.02465600 -1.78465000 0.70575400

N 0.18539300 0.04875700 2.21930600

N 2.61910300 1.44359000 -0.62894600

N 1.32233100 -0.05645200 -2.30548300

N 2.85331500 -1.14988900 -0.50567700

N 0.90882800 -2.20113900 -0.91675000

N 1.40467500 -1.72565700 1.58524200

N 2.62676500 0.56444300 1.44188200

B -0.42606600 -1.68441200 -0.62415100

N 0.71289000 2.23746600 0.94459700

N -1.35054500 1.51322700 0.59312300

Pd -2.44165700 -0.04591200 -0.09775800

H -3.68165000 0.06540600 1.38569700

H -3.96058400 0.61248300 0.91219200

b [B_11_N_12_Pd(2H)]

B 0.10970900 1.94563800 0.83019100

B 0.23454700 1.72018700 -1.08348100

B -0.00440500 -0.29321500 1.99168500

B -2.07989400 1.76549700 -0.27120300

B -1.70538200 0.66613400 1.82519200

B -1.79874400 -1.61575100 1.07980100

B -2.98085000 0.08812000 -0.14087000

B -1.68738200 -1.83328800 -0.81269100

B 0.21128700 -0.73216600 -1.80072400

B -1.47188800 0.23351100 -2.00776800

N -0.31485900 1.19922900 1.95610300

N 1.02607900 -1.02119800 1.38469400

N 1.16405700 -1.40136600 -0.98898300

N -0.05715200 0.72620900 -2.05940400

N -2.71073800 1.13340800 0.89341600

N -1.43611800 -0.73215400 2.15583000

N -2.81333200 -1.32058200 0.02288000

N -0.84998500 -2.39154300 0.25870600

N -1.19252300 -1.20653400 -2.01707400

N -2.57200500 0.88494300 -1.34186300

B 0.49199800 -1.78606500 0.28248200

N -0.77648000 2.39221200 -0.25915300

N 1.36496500 1.78334700 -0.04707200

Pd 2.43936800 -0.08911900 0.04497400

H 3.35827100 0.41941600 -1.11912100

H 2.01249300 2.56526300 -0.11929100

c (B_11_N_12_Pd-C_2_H_2_)

B -0.23546400 1.86761100 -0.92299100

B -0.23573400 1.86642100 0.92477700

B -0.15798500 -0.46726600 -1.88653600

B 2.03225700 1.78937100 0.00111300

B 1.52741500 0.45361800 -1.92337800

B 1.66042400 -1.75024300 -0.95635600

B 2.91299000 0.07860200 0.00025300

B 1.66030300 -1.75136600 0.95459300

B -0.15833500 -0.46971700 1.88592200

B 1.52702400 0.45139500 1.92396100

N 0.13442000 0.97186300 -2.00694800

N -1.17563200 -1.20594200 -1.17300500

N -1.17592000 -1.20768600 1.17165700

N 0.13395700 0.96939400 2.00772600

N 2.59180100 0.99887200 -1.12350800

N 1.22682900 -0.98760400 -2.09820000

N 2.72576300 -1.34743800 -0.00058300

N 0.76170400 -2.44052900 -0.00135700

N 1.22654100 -0.99003300 2.09724700

N 2.59159000 0.99761200 1.12497300

B -0.55269800 -1.83318600 -0.00106100

N 0.77195100 2.45957900 0.00137200

N -1.37590800 1.82568200 0.00073700

Pd -2.36871000 -0.03223000 -0.00004900

C -4.27447439 1.18319457 -0.00666572

C -4.41480618 -0.01495973 0.05671077

H -4.15059361 2.24089002 -0.06261263

H -4.53868695 -1.07265518 0.11265767

d (B_11_N_12_Pd-CCH_2_)

B 0.34396500 -2.04012500 0.40431600

B 0.48865700 -1.50674700 -1.37971900

B -0.00907800 -0.09179900 1.80865900

B 2.63911900 -1.49689900 -0.20551800

B 1.77699400 -0.76622200 1.91595600

B 1.66474700 1.60057200 1.45348000

B 3.28138500 0.23809200 0.31502100

B 1.90506600 2.08926700 -0.38192100

B 0.42259200 0.98362000 -1.89017600

B 2.19451000 0.21389200 -1.82373400

N 0.48777600 -1.48141700 1.73313700

N -0.99469900 0.59708200 1.02505900

N -0.75033000 1.51780000 -1.26714200

N 0.88110400 -0.38123200 -2.20749700

N 2.97581500 -0.96177200 1.13908600

N 1.23484400 0.54908200 2.32955000

N 2.89460200 1.59201200 0.61409400

N 0.78125600 2.35893300 0.54616500

N 1.76701100 1.63041700 -1.74181100

N 3.21606900 -0.40670300 -1.02748400

B -0.41767900 1.60291700 0.15479500

N 1.47332700 -2.25565100 -0.53541400

N -0.70827200 -1.76619700 -0.57459600

Pd -2.24453900 -0.58175000 -0.13014800

H -4.58556100 2.45906700 -0.03456000

C -4.70809500 1.43888000 0.33188000

C -3.75552600 0.57020300 0.18727000

H -5.64485700 1.16761600 0.81866700

Figure 3

IS

B -0.22220400 2.02955900 -0.19150900

B -0.23597300 1.23743300 1.51710800

B -0.06575800 0.31693300 -1.91112300

B 2.01697300 1.69474100 0.70436500

B 1.59047900 1.22655400 -1.61413400

B 1.77944300 -1.18140800 -1.53567700

B 2.98283400 0.16985400 0.05029900

B 1.82017800 -1.93822200 0.22282300

B -0.02915000 -1.28103900 1.59893500

B 1.60964900 -0.31099400 1.96123000

N 0.19052200 1.72313700 -1.53870900

N -1.04055700 -0.60725100 -1.40746100

N -1.02465600 -1.78465000 0.70575400

N 0.18539300 0.04875700 2.21930600

N 2.61910300 1.44359000 -0.62894600

N 1.32233100 -0.05645200 -2.30548300

N 2.85331500 -1.14988900 -0.50567700

N 0.90882800 -2.20113900 -0.91675000

N 1.40467500 -1.72565700 1.58524200

N 2.62676500 0.56444300 1.44188200

B -0.42606600 -1.68441200 -0.62415100

N 0.71289000 2.23746600 0.94459700

N -1.35054500 1.51322700 0.59312300

Pd -2.44165700 -0.04591200 -0.09775800

H -3.68165000 0.06540600 1.38569700

H -3.96058400 0.61248300 0.91219200

TS

B -0.23937600 1.82462400 -0.99452000

B -0.19333400 1.88157200 0.88971700

B -0.13482400 -0.57352900 -1.85537600

B 2.04714000 1.80674500 -0.08492900

B 1.53123200 0.40309200 -1.95015100

B 1.73025400 -1.75741800 -0.90543400

B 2.96237100 0.12487400 -0.03821100

B 1.76141500 -1.69019800 1.00360700

B -0.07795100 -0.43077300 1.92696700

B 1.59716000 0.53733900 1.89645500

N 0.12123200 0.88854400 -2.02899700

N -1.11505700 -1.28030800 -1.09504200

N -1.08043500 -1.21782000 1.27576800

N 0.19082500 1.03122500 1.98665200

N 2.60029900 1.00052100 -1.19079100

N 1.26635200 -1.04313100 -2.06856900

N 2.80655500 -1.29938300 0.01626000

N 0.86224800 -2.42375100 0.08984900

N 1.33349000 -0.89483800 2.12670800

N 2.63715000 1.08045100 1.05908400

B -0.47427400 -1.84325100 0.09250300

N 0.76339600 2.44318300 -0.08846200

N -1.36392700 1.86009400 -0.02370400

Pd -2.38541000 -0.11016700 -0.03715400

H -3.35475100 1.03437100 0.89040600

H -2.62034300 1.60443500 0.50423900

FS

B 0.10970900 1.94563800 0.83019100

B 0.23454700 1.72018700 -1.08348100

B -0.00440500 -0.29321500 1.99168500

B -2.07989400 1.76549700 -0.27120300

B -1.70538200 0.66613400 1.82519200

B -1.79874400 -1.61575100 1.07980100

B -2.98085000 0.08812000 -0.14087000

B -1.68738200 -1.83328800 -0.81269100

B 0.21128700 -0.73216600 -1.80072400

B -1.47188800 0.23351100 -2.00776800

N -0.31485900 1.19922900 1.95610300

N 1.02607900 -1.02119800 1.38469400

N 1.16405700 -1.40136600 -0.98898300

N -0.05715200 0.72620900 -2.05940400

N -2.71073800 1.13340800 0.89341600

N -1.43611800 -0.73215400 2.15583000

N -2.81333200 -1.32058200 0.02288000

N -0.84998500 -2.39154300 0.25870600

N -1.19252300 -1.20653400 -2.01707400

N -2.57200500 0.88494300 -1.34186300

B 0.49199800 -1.78606500 0.28248200

N -0.77648000 2.39221200 -0.25915300

N 1.36496500 1.78334700 -0.04707200

Pd 2.43936800 -0.08911900 0.04497400

H 3.35827100 0.41941600 -1.11912100

H 2.01249300 2.56526300 -0.11929100

Figure 4

R1

Co-ads

B 0.10970900 1.94563800 0.83019100

B 0.23454700 1.72018700 -1.08348100

B -0.00440500 -0.29321500 1.99168500

B -2.07989400 1.76549700 -0.27120300

B -1.70538200 0.66613400 1.82519200

B -1.79874400 -1.61575100 1.07980100

B -2.98085000 0.08812000 -0.14087000

B -1.68738200 -1.83328800 -0.81269100

B 0.21128700 -0.73216600 -1.80072400

B -1.47188800 0.23351100 -2.00776800

N -0.31485900 1.19922900 1.95610300

N 1.02607900 -1.02119800 1.38469400

N 1.16405700 -1.40136600 -0.98898300

N -0.05715200 0.72620900 -2.05940400

N -2.71073800 1.13340800 0.89341600

N -1.43611800 -0.73215400 2.15583000

N -2.81333200 -1.32058200 0.02288000

N -0.84998500 -2.39154300 0.25870600

N -1.19252300 -1.20653400 -2.01707400

N -2.57200500 0.88494300 -1.34186300

B 0.49199800 -1.78606500 0.28248200

N -0.77648000 2.39221200 -0.25915300

N 1.36496500 1.78334700 -0.04707200

Pd 2.43936800 -0.08911900 0.04497400

H 3.35827100 0.41941600 -1.11912100

H 2.01249300 2.56526300 -0.11929100

C 3.08701624 1.46509852 2.02624200

C 3.23472818 0.30075604 1.74028557

H 2.95662047 2.49294589 2.27867595

H 3.36512395 -0.72709133 1.48785162

TS1

B -0.05811100 1.92451400 0.67587100

B -0.17198200 1.67305700 -1.24086300

B -0.12425600 -0.26216800 1.93343500

B -2.36804000 1.83048100 -0.15312600

B -1.79682600 0.76229800 1.91483200

B -2.05877500 -1.52567900 1.24357600

B -3.30936400 0.19656800 0.12094900

B -2.16699300 -1.77844800 -0.64533800

B -0.34903100 -0.75866500 -1.86079600

B -2.02379800 0.25002800 -1.92073200

N -0.37796500 1.23741600 1.87623500

N 0.80556600 -1.07267900 1.27154000

N 0.65932200 -1.38162200 -1.10915600

N -0.61892400 0.69961100 -2.17339300

N -2.88423600 1.25136900 1.09424900

N -1.54802600 -0.63777400 2.25520800

N -3.17698900 -1.21390500 0.30145800

N -1.23479600 -2.34715200 0.34105400

N -1.78894300 -1.19449100 -1.90648800

N -3.01647800 0.95256800 -1.13742200

B 0.11873000 -1.78241800 0.19541100

N -1.04821500 2.39885700 -0.30652700

N 1.05800400 1.66718800 -0.33681900

Pd 2.18802800 -0.30926800 -0.15559600

H 3.16332500 0.05550100 -1.39170700

H 1.79089500 2.35776900 -0.47258600

C 4.33867200 0.48197100 -0.13498400

C 3.85767000 0.43250700 1.01907700

H 5.09793700 0.63908800 -0.87648300

H 3.86450000 0.52357100 2.08796200

IMS1

B -0.02271800 0.96709900 -1.74493400

B -0.36086400 1.85194900 -0.14375200

B 0.70662500 -1.45170200 -1.49361600

B 2.01276500 1.91761000 -0.76802000

B 2.14900600 -0.22688700 -1.81620500

B 2.46387300 -1.61454600 0.11009100

B 3.18183800 0.68866800 0.13921300

B 2.10954800 -0.71836500 1.77373600

B -0.03811500 0.35202400 1.85680800

B 1.42243000 1.56924700 1.52948700

N 0.71606100 -0.19189900 -2.23072000

N -0.33985300 -2.04682400 -0.62743700

N -0.75393300 -0.82705200 1.48647000

N -0.02988400 1.71728000 1.26671300

N 2.91900300 0.87107000 -1.31642000

N 2.16557800 -1.62436100 -1.30127700

N 3.24823200 -0.57699200 0.82498000

N 1.50787700 -1.94840800 1.19098000

N 1.37044800 0.35242700 2.37099300

N 2.50020300 1.91085200 0.63192200

B 0.11943000 -1.73890400 0.81848000

N 0.67381500 2.16239200 -1.18359600

N -1.26898100 1.09389900 -1.00405600

Pd -2.12752900 -0.58320000 -0.14628400

H -3.68292600 0.23196300 1.72108100

H -0.48332000 -3.03974300 -0.81292200

H -5.12041200 1.87692700 0.48747100

C -4.23832500 1.43277600 0.02418300

C -3.51917700 0.52837100 0.68328600

H -3.98106800 1.76355000 -0.97771600

TS2

B 0.21563700 1.82829600 0.60947000

B 0.13114400 1.55138200 -1.26377200

B -0.20643800 -0.27932000 1.95212100

B -2.06026900 2.03169000 -0.25080600

B -1.70066500 0.95398300 1.85900700

B -2.27773400 -1.30094500 1.26583200

B -3.23426900 0.55087300 0.05113200

B -2.37639900 -1.59835100 -0.61832100

B -0.41259600 -0.87030100 -1.79674500

B -1.90430600 0.35403200 -1.94625300

N -0.22906300 1.20825800 1.82779300

N 0.61633600 -1.29179100 1.37476100

N 0.48747900 -1.61374200 -0.98624100

N -0.44428600 0.58466600 -2.15957000

N -2.68763500 1.56265600 1.00168700

N -1.66295900 -0.46153500 2.26369200

N -3.30842300 -0.86158300 0.28175600

N -1.56294600 -2.27203200 0.41549400

N -1.88414400 -1.11766600 -1.88473400

N -2.80984800 1.20990200 -1.21938900

B -0.14760500 -1.92303100 0.28886100

N -0.68987500 2.43064100 -0.39128200

N 1.30071800 1.47840800 -0.34622700

Pd 2.02300500 -0.54968400 -0.15506300

H 4.71531000 0.84572000 -1.12647400

H 2.52652300 1.30243600 0.08793700

C 4.74533700 0.82722300 -0.03666600

C 3.63359500 0.67302000 0.69969300

H 5.73236200 0.96275700 0.40809900

H 3.68400800 0.68828900 1.78837900

IMS2

B 0.16113400 1.26160900 1.34840500

B 0.00459100 1.96371300 -0.39217500

B -0.18365900 -1.23158700 1.60120200

B -2.13713000 1.82045000 0.75944800

B -1.71054600 -0.13880500 2.08016100

B -2.20464000 -1.83573400 0.47080400

B -3.25687400 0.32931800 0.30376800

B -2.31651000 -1.17438700 -1.32143500

B -0.43908400 0.17402400 -1.97924900

B -1.99751900 1.20730600 -1.55965400

N -0.24677200 0.14052500 2.16238000

N 0.67440200 -1.84953800 0.64194900

N 0.53304800 -0.76891200 -1.52459700

N -0.57229300 1.61704000 -1.66542300

N -2.72655600 0.77173700 1.62497300

N -1.63236000 -1.58178100 1.76942600

N -3.26726100 -1.02256900 -0.18485700

N -1.44067700 -2.21194100 -0.74021400

N -1.88573500 -0.12196700 -2.19994800

N -2.89769200 1.54094100 -0.48291600

B -0.04929100 -1.76248500 -0.62956600

N -0.78531300 2.29005400 0.82594900

N 1.18162400 1.42383600 0.30103200

Pd 2.13757200 -0.20016700 -0.41721200

H 4.01333000 1.72189700 0.17200800

H 3.18141600 0.35961600 2.08014700

C 4.20105100 0.65402200 0.22215300

C 3.74059700 -0.09415800 1.26814800

H 4.89341800 0.23839400 -0.50683800

H 4.04034500 -1.13047800 1.40179000

R2

Co-ads

B 0.25590300 -1.80521700 1.00274000

B 0.54588000 -1.87876200 -0.83501600

B -0.12477500 0.50843800 1.66538400

B 2.60858300 -1.50120000 0.43175500

B 1.62323500 -0.11376000 2.12421400

B 1.62277400 1.97248200 0.90870200

B 3.27248700 0.30193500 0.40817300

B 2.01103500 1.83334500 -0.96144000

B 0.60775700 0.30811900 -2.13921700

B 2.33992100 -0.41261400 -1.68315400

N 0.32613400 -0.83631700 2.08454300

N -1.03331700 0.89987100 0.62269600

N -0.58519600 1.03189100 -1.82441700

N 1.03738700 -1.08791000 -1.95354400

N 2.86978500 -0.55863300 1.55170600

N 1.09795800 1.27098200 2.04465800

N 2.90780000 1.68293500 0.21797700

N 0.83018900 2.39750200 -0.26348400

N 1.95913400 0.95754300 -2.10520000

N 3.28295600 -0.74422700 -0.65205200

B -0.36027800 1.56340800 -0.48093300

N 1.45187000 -2.31897600 0.27779100

N -0.70505200 -1.87914600 -0.08617300

Pd -2.27064700 -0.59443200 -0.10143300

H -3.63209300 2.79567500 0.89928600

C -4.15057700 1.95642100 0.43847300

C -3.53354800 0.85275300 0.14827500

H -5.21300100 2.05102500 0.21326300

H -3.28555900 -1.84018800 -1.14768700

H -3.40496800 -2.10717900 -0.42699500

TS1

B -0.22817900 1.40975000 -1.24127100

B -0.17892400 1.77713300 0.58475900

B 0.34198800 -0.98649300 -1.65524100

B 2.01557800 2.03229100 -0.48633700

B 1.77527100 0.22988800 -2.04295300

B 2.41626100 -1.59353600 -0.60485300

B 3.27642500 0.61270800 -0.19654300

B 2.51316700 -1.15034000 1.25765800

B 0.51856200 -0.15217800 2.11570800

B 1.91947700 1.11432100 1.71732900

N 0.30161500 0.40756200 -2.15586300

N -0.46973600 -1.64972900 -0.66055200

N -0.28121300 -1.29580900 1.79651000

N 0.43987200 1.28686800 1.80883500

N 2.71277100 1.15530800 -1.46172800

N 1.79447700 -1.23877500 -1.84947200

N 3.41906700 -0.78165600 0.13491900

N 1.71184200 -2.18036300 0.55493000

N 2.00736700 -0.25822400 2.26959500

N 2.79361100 1.67745100 0.72567800

B 0.28544400 -1.86024000 0.57666100

N 0.63897500 2.38706300 -0.51757100

N -1.27866700 1.31035500 -0.24287700

Pd -2.24985600 -0.49533300 -0.17258000

H -2.76851400 -2.15615300 -0.36266700

H -1.83032300 -2.17975900 -0.60435800

H -5.78158000 0.95311900 0.68134200

C -4.76531000 1.31320600 0.53031900

C -3.79733400 0.50037700 0.24703800

H -4.55556200 2.37973000 0.61879500

IMS1

B -0.02271800 0.96709900 -1.74493400

B -0.36086400 1.85194900 -0.14375200

B 0.70662500 -1.45170200 -1.49361600

B 2.01276500 1.91761000 -0.76802000

B 2.14900600 -0.22688700 -1.81620500

B 2.46387300 -1.61454600 0.11009100

B 3.18183800 0.68866800 0.13921300

B 2.10954800 -0.71836500 1.77373600

B -0.03811500 0.35202400 1.85680800

B 1.42243000 1.56924700 1.52948700

N 0.71606100 -0.19189900 -2.23072000

N -0.33985300 -2.04682400 -0.62743700

N -0.75393300 -0.82705200 1.48647000

N -0.02988400 1.71728000 1.26671300

N 2.91900300 0.87107000 -1.31642000

N 2.16557800 -1.62436100 -1.30127700

N 3.24823200 -0.57699200 0.82498000

N 1.50787700 -1.94840800 1.19098000

N 1.37044800 0.35242700 2.37099300

N 2.50020300 1.91085200 0.63192200

B 0.11943000 -1.73890400 0.81848000

N 0.67381500 2.16239200 -1.18359600

N -1.26898100 1.09389900 -1.00405600

Pd -2.12752900 -0.58320000 -0.14628400

H -3.68292600 0.23196300 1.72108100

H -0.48332000 -3.03974300 -0.81292200

H -5.12041200 1.87692700 0.48747100

C -4.23832500 1.43277600 0.02418300

C -3.51917700 0.52837100 0.68328600

H -3.98106800 1.76355000 -0.97771600

TS2

B 0.06842500 0.40789100 -1.96741600

B -0.29274700 1.73165000 -0.72364700

B 0.72585400 -1.84116900 -0.98467700

B 2.09498000 1.57926700 -1.23868300

B 2.21076300 -0.78282700 -1.59037200

B 2.41198200 -1.53422500 0.67093200

B 3.19372000 0.66139500 0.04589800

B 2.01634900 -0.17584200 1.97012200

B -0.09448600 0.90669500 1.62191200

B 1.41044700 1.95392300 1.03036500

N 0.79646200 -0.85308000 -2.05727100

N -0.36470700 -2.15561500 -0.03274900

N -0.82798000 -0.30794500 1.55344400

N -0.02950300 2.05404500 0.67244800

N 2.99216700 0.40377000 -1.40767100

N 2.17440700 -1.96446900 -0.68528700

N 3.19824800 -0.34161300 1.08024500

N 1.40158300 -1.51150900 1.75317300

N 1.28740000 1.03887400 2.18584700

N 2.52981000 1.98758400 0.11975800

B 0.03478200 -1.40164400 1.25707400

N 0.78059800 1.70865200 -1.76726100

N -1.19070600 0.75648000 -1.33390500

Pd -2.18514500 -0.63497000 -0.13044300

C -3.74120700 0.44230400 0.15822800

C -3.83433800 1.80729100 0.31478900

H -3.88255500 0.88103700 1.33650400

H -4.81872100 2.25621700 0.48042900

H -2.99357700 2.50331900 0.21694200

H -0.46999500 -3.16131500 0.10546000

IMS2

B -0.23928100 1.25023300 -1.31559800

B -0.27707200 1.84336200 0.45838500

B 0.50769100 -1.18156500 -1.72140300

B 1.94253600 2.05677400 -0.55084700

B 1.87331200 0.15757100 -2.00218700

B 2.50284400 -1.56920000 -0.48872700

B 3.23339800 0.70314100 -0.11478000

B 2.41752300 -1.02165100 1.34936500

B 0.33425800 -0.00130500 1.95860100

B 1.74941800 1.27013900 1.70625900

N 0.38686500 0.22320600 -2.12936200

N -0.34604700 -2.04580900 -0.89173300

N -0.45635100 -1.01151700 1.35134100

N 0.27897800 1.47234400 1.74057300

N 2.72643100 1.16238000 -1.44071600

N 1.98873900 -1.31074200 -1.81556200

N 3.39912500 -0.66919800 0.28490900

N 1.72368900 -2.12498700 0.64280300

N 1.79751000 -0.10136100 2.25635500

N 2.66370300 1.79436500 0.71704300

B 0.28894800 -1.86400100 0.49108300

N 0.55200600 2.34692100 -0.67941100

N -1.33775300 1.24196000 -0.35541100

Pd -2.17940300 -0.56358100 0.19665400

C -3.81346300 0.14488400 -0.12245600

C -4.58211400 1.34448800 -0.38973400

H -5.10144300 1.67603500 0.52211200

H -5.35182200 1.11377700 -1.14211900

H -3.93175300 2.15218500 -0.75688400

H -0.29512600 -3.01393600 -1.21259600

TS3

B 0.34262800 1.74646600 -0.43716900

B -0.02303700 0.68769600 -1.93507700

B 0.08573400 0.57665700 1.80520100

B -2.02155100 1.76209400 -1.01451400

B -1.38462100 1.73917600 1.29954600

B -2.06463900 -0.48316400 1.88979600

B -3.16096300 0.67555200 0.08063600

B -2.42291800 -1.59630200 0.37901100

B -0.64683000 -1.60773200 -1.24392200

B -2.14907600 -0.51394400 -1.75674300

N 0.07387100 1.82766000 0.98460100

N 0.82207200 -0.64010000 1.70506100

N 0.37203400 -1.88819600 -0.29450000

N -0.74785000 -0.51581300 -2.25459900

N -2.47200800 1.96445300 0.38267400

N -1.31810400 0.67458500 2.31853000

N -3.21755800 -0.46827300 0.94628000

N -1.47341100 -1.77805600 1.49715500

N -2.11703000 -1.78800700 -1.01208400

N -2.92281700 0.64990600 -1.38916700

B -0.08534600 -1.60480800 1.05850700

N -0.68483100 1.94990200 -1.49002600

N 1.25043800 0.89083400 -1.22157700

Pd 2.04440100 -0.70619500 -0.15059900

C 3.74911000 0.46786100 0.12927200

C 3.76938200 1.76649200 0.59842700

H 4.68704900 2.35353200 0.68090500

H 2.84642700 2.26183000 0.90023900

H 3.28683400 1.31336400 -0.66594200

H 4.72572000 0.08941200 -0.18662000

IMS3

B 0.36400500 1.75099500 -0.39795900

B -0.04744800 0.71522900 -1.91110400

B 0.11229200 0.52203700 1.79415400

B -2.00766400 1.80931900 -0.94792600

B -1.34528000 1.71279100 1.35754100

B -2.04635000 -0.52321000 1.88604200

B -3.15145400 0.70967300 0.13130700

B -2.45214800 -1.58421900 0.34865000

B -0.69948000 -1.58776900 -1.29885400

B -2.18952000 -0.44532800 -1.75466900

N 0.10738300 1.79597400 1.02750800

N 0.82802500 -0.70889000 1.62360000

N 0.33408800 -1.90951000 -0.38374600

N -0.79508500 -0.45838000 -2.27023300

N -2.43900700 1.97818900 0.46180000

N -1.27506800 0.60880900 2.33999100

N -3.21369100 -0.45897000 0.96394100

N -1.48383100 -1.81397800 1.44509300

N -2.17285000 -1.74171900 -1.05099200

N -2.93247900 0.72320800 -1.34068900

B -0.10773800 -1.65905400 0.97079700

N -0.67266900 1.98617300 -1.43551800

N 1.23691700 0.89036300 -1.20648000

Pd 2.03568800 -0.69090100 -0.15774800

C 3.66450900 0.31583100 0.16172600

C 3.86338400 1.73826400 0.45582100

H 4.75919300 1.92962400 1.06042600

H 2.98371800 2.22672600 0.87997100

H 4.05187800 2.21308400 -0.52734300

H 4.59797900 -0.25374600 0.02823700

Figure 5

R3

Co-ads

B -0.06927100 1.84816600 -0.60580800

B -0.08877000 1.38472400 1.20982000

B 0.36866400 -0.13948300 -1.92527900

B 2.14425900 1.96088400 0.40581900

B 1.89442400 1.00994300 -1.78518900

B 2.35487900 -1.29212700 -1.22084800

B 3.31774600 0.47293200 0.09322400

B 2.39906800 -1.68639700 0.65039600

B 0.43535400 -1.02868500 1.83165800

B 1.91261700 0.20487100 2.01838000

N 0.44771800 1.34187200 -1.85527600

N -0.51904200 -1.03436100 -1.25707900

N -0.42997300 -1.86399000 1.05810300

N 0.43916300 0.41447600 2.14902100

N 2.84346400 1.53627200 -0.83257500

N 1.81187800 -0.40463700 -2.20854100

N 3.37224700 -0.93622300 -0.19206400

N 1.57142300 -2.26390500 -0.43173600

N 1.90839400 -1.27416400 1.94419400

N 2.84757500 1.08165600 1.37135900

B 0.16224500 -1.86219600 -0.28750000

N 0.77975500 2.37429000 0.49951100

N -1.16648100 1.34765600 0.21938200

Pd -2.19996200 -0.27500800 -0.31963100

C -4.16896300 0.90378000 0.11140000

C -3.80695500 0.29190200 1.27799900

H -3.83818900 1.91243600 -0.11382500

H -4.91401900 0.46721500 -0.54871400

H -4.24741100 -0.65301300 1.58471600

H -3.18838000 0.80359700 2.00776200

H -3.20458200 -1.65986900 -1.21078900

H -3.05261500 -1.98983500 -0.52595300

TS1

B -0.54867500 1.93211400 1.04855600

B -0.21355000 1.90117900 -0.80842100

B -0.78501100 -0.42625700 2.07626600

B -2.61917400 1.77047900 -0.26772400

B -2.44655700 0.51091100 1.74687900

B -2.39342300 -1.70494500 0.84563200

B -3.47691300 0.06546700 -0.37644200

B -2.04415900 -1.74855700 -1.02995700

B -0.09430000 -0.49944600 -1.66615000

B -1.75860600 0.42295600 -2.02977700

N -1.07382400 1.03114300 2.05156800

N 0.30269200 -1.25813100 1.67064600

N 0.76494800 -1.17384800 -0.75512200

N -0.36706300 0.96149100 -1.89511700

N -3.36593600 1.02275500 0.76496100

N -2.20289100 -0.91304800 2.03957600

N -3.28215400 -1.35303300 -0.29920400

N -1.31834500 -2.37414800 0.09232300

N -1.42949100 -1.00715600 -2.09874200

N -2.96574600 0.96507100 -1.45215100

B -0.00194900 -1.75239900 0.32318400

N -1.38393700 2.44464000 -0.06390200

N 0.71984800 2.03515100 0.32133100

Pd 2.50599200 -0.30168100 -0.17176900

H 3.16048300 -1.60690300 -0.53929400

H 1.64370800 1.70192100 0.55720300

C 4.53991200 0.67881400 -0.29653700

C 4.29211000 0.39415200 1.02491300

H 4.34333600 1.66615400 -0.70804700

H 5.13267700 0.01365500 -0.91661800

H 4.67131300 -0.51029200 1.49100200

H 3.90562500 1.15128400 1.70349500

IMS1

B 0.05536700 -1.81145200 0.46055300

B -0.19749500 -0.54739900 1.85584600

B -0.15990900 -0.69280000 -1.67588300

B -2.28330700 -1.63678800 1.12415500

B -1.68835400 -1.77880300 -1.20903200

B -2.23360600 0.48498200 -1.82983400

B -3.38977100 -0.54986800 -0.00177700

B -2.57823900 1.68360200 -0.38279100

B -0.84565600 1.84942600 1.22735800

B -2.30601300 0.67880800 1.75839500

N -0.24651000 -1.99704200 -0.92761700

N 0.61741800 0.45712800 -1.37206300

N 0.14601500 2.28751800 0.29650600

N -0.86651100 0.68622800 2.17584800

N -2.76880200 -1.88278700 -0.25062200

N -1.55179400 -0.71264600 -2.21716700

N -3.40595600 0.56054000 -0.90853000

N -1.57611500 1.74727200 -1.46275600

N -2.30639600 1.94444700 1.00685700

N -3.11811100 -0.47282100 1.46910700

B -0.19227500 1.59263000 -0.97319200

N -0.92338900 -1.81710400 1.56362300

N 1.03420300 -0.86901000 1.07322100

Pd 2.39812100 0.08701200 -0.30909100

C 4.49505400 -0.61278900 0.11597000

C 4.13464900 0.25062500 1.12376700

H 4.37279700 -1.68702400 0.22698200

H 5.10207000 -0.27841300 -0.71920700

H 4.43847900 1.29313700 1.10029600

H 3.72004600 -0.11845000 2.05663900

H 3.03595500 1.00377100 -1.36647700

H 1.09680100 2.07365700 0.58768100

TS2

B 0.31273800 1.78280800 -0.58125900

B -0.02333900 0.55218100 -1.97849300

B 0.04493400 0.75731300 1.72345700

B -2.05616000 1.67711300 -1.16715500

B -1.43236000 1.86891800 1.13756300

B -2.11197100 -0.29666700 1.92447400

B -3.19832500 0.69004000 0.00987700

B -2.46600400 -1.54691400 0.52477800

B -0.66759000 -1.72850200 -1.05702200

B -2.14786700 -0.65350900 -1.68816000

N 0.02460900 1.94486000 0.81869600

N 0.77150700 -0.47082600 1.70196000

N 0.32323500 -1.99916000 -0.07375500

N -0.72502500 -0.69365600 -2.14008200

N -2.51771800 2.00425600 0.19787200

N -1.35837300 0.89184200 2.23724500

N -3.25929800 -0.36810900 0.97475500

N -1.53010300 -1.62759200 1.66633800

N -2.14070900 -1.86097300 -0.84384400

N -2.93836000 0.52937800 -1.45120000

B -0.13966900 -1.52062700 1.22316200

N -0.71285000 1.82578800 -1.64500000

N 1.25933600 0.89761400 -1.30819900

Pd 1.95971300 -0.77899600 -0.13570200

C 3.77631700 0.61743500 -0.27379400

C 3.90161000 1.60095200 0.88501600

H 4.94151800 1.93250500 1.00580500

H 3.58345500 1.15131200 1.82997700

H 3.29214800 2.49583600 0.72068400

H 4.15377900 1.03484100 -1.21876600

H 2.51682500 0.78833700 -0.94783900

H 4.35222100 -0.30405400 -0.11449100

IMS2

B 0.29562100 1.57805600 -0.80060300

B -0.17576600 0.25003700 -2.04132600

B 0.06231400 0.96074400 1.64266400

B -2.08499700 1.58859800 -1.33106300

B -1.38366600 2.03624100 0.92283700

B -2.12102100 0.02960000 1.99514400

B -3.23120200 0.81055800 -0.00055900

B -2.56142400 -1.37045600 0.76718300

B -0.85758000 -1.78733800 -0.88333400

B -2.33741400 -0.78887900 -1.56840000

N 0.06232300 1.98477300 0.56544600

N 0.75357500 -0.28003800 1.79412600

N 0.18916000 -1.88973800 0.09520500

N -0.95944900 -0.96128400 -2.10120000

N -2.48689300 2.10042500 -0.00058000

N -1.32682600 1.21949100 2.15244300

N -3.29809000 -0.11939800 1.09402400

N -1.58070000 -1.34591500 1.87059400

N -2.30974200 -1.87251700 -0.55721000

N -3.04375100 0.45918700 -1.43518900

B -0.20435900 -1.30152100 1.38571400

N -0.75434300 1.60694100 -1.86097600

N 1.11496200 0.50337800 -1.39160100

Pd 1.89266800 -0.86427200 -0.11785600

C 4.38007200 0.35285100 0.21575400

C 4.66401300 1.85744600 0.16982400

H 5.38423500 2.13335700 0.94689700

H 3.75203500 2.43811000 0.33553900

H 5.08071200 2.15214400 -0.79798000

H 5.27659000 -0.25161500 0.04581000

H 3.69431300 0.09637000 -0.62444700

H 3.96075700 0.06104800 1.18520500
